# EEG Neurofeedback for Attention and Executive Functions in Intellectual Disability: A Systematic Review of Treatment Studies

**DOI:** 10.3390/jcm15134947

**Published:** 2026-06-25

**Authors:** Marilena Recupero, Raffaele Ferri, Serafino Buono

**Affiliations:** 1 Oasi Research Institute—IRCCS, 94018 Troina, Italy; rferri@oasi.en.it (R.F.); fbuono@oasi.en.it (S.B.); 2 Department of Medicine and Surgery, “Kore” University of Enna, 94100 Enna, Italy

**Keywords:** neurofeedback, EEG biofeedback, intellectual disability, attention, executive functions, self-regulation, neurodevelopmental disorders

## Abstract

**Background:** EEG neurofeedback has been proposed as a non-pharmacological approach to enhance attention and cognitive performance in neurodevelopmental conditions. However, its efficacy in individuals with intellectual disability (ID) remains limited. **Methods:** We conducted a systematic review of treatment studies evaluating EEG-based neurofeedback targeting attention and executive function outcomes in participants with ID. Searches were performed in Embase, Web of Science, Scopus, PubMed, and the Cochrane Library from inception to 12 March 2026. Two reviewers independently completed screening, eligibility assessment, data extraction, and risk-of-bias appraisal, with disagreements resolved by a third reviewer. Due to heterogeneity in study designs, neurofeedback protocols, participant characteristics, and outcome measures, data were synthesized narratively. **Results:** Six studies met the inclusion criteria, comprising small randomized or controlled experimental studies, case series, and single-case reports. Several studies suggested improvements in attention-related neuropsychological performance and behavioral or task-based outcomes, and in some cases were accompanied by parallel EEG changes. Nevertheless, the overall body of evidence was constrained by small sample sizes, heterogeneous populations, limited blinding, infrequent sham or active controls, variable follow-up durations, and incomplete effect-size reporting. **Conclusions:** EEG neurofeedback appears to confer benefits for attention-related outcomes in individuals with ID, but current findings are preliminary and warrant larger, well-controlled trials with standardized protocols and reporting.

## 1. Introduction

Attention refers to the capacity of the cognitive system to regulate selectivity and intensity in the processing of information. The selective dimension allows individuals to prioritize relevant stimuli while suppressing competing information, whereas the intensity dimension supports alertness, sustained attention, and readiness to respond over time [1]. These attentional mechanisms are closely linked to executive functions (EFs), since early attentional control contributes to the development of self-regulation, inhibitory control, working memory, and goal-directed behavior [2,3].

Executive functions comprise a set of higher-order cognitive processes that enable individuals to plan, monitor, inhibit inappropriate responses, shift flexibly between tasks, regulate behavior, and maintain information in working memory [4,5,6]. Deficits in attention and EFs may substantially affect learning, adaptive functioning, autonomy, social participation, and quality of life. These difficulties are especially relevant in individuals with intellectual disability (ID), in whom cognitive limitations are often accompanied by impairments in adaptive behavior, reduced self-regulatory capacity, and frequent neurodevelopmental or neurological comorbidities.

Neurofeedback (NFB) is a form of biofeedback that aims to train self-regulation of brain activity by providing real-time feedback derived from electrophysiological signals [7]. In EEG neurofeedback, individuals receive contingent visual, auditory, or game-like feedback when selected EEG features move in a desired direction, such as increased sensorimotor rhythm (SMR), increased beta activity, reduced theta/beta ratio, or suppression of excessive alpha or theta activity. Through repeated training, participants are expected to acquire greater voluntary modulation of specific EEG patterns, which may in turn influence attention, arousal regulation, and behavioral control.

NFB has been explored in several neurodevelopmental and cognitive conditions, including attention-deficit/hyperactivity disorder (ADHD), learning difficulties, and other developmental disorders [8,9,10,11,12,13]. However, evidence from these populations cannot be directly generalized to individuals with ID. Individuals with ID may differ in baseline cognitive profile, learning capacity, adaptive functioning, communication abilities, sensory tolerance, task comprehension, and comorbid neurological or psychiatric conditions. These factors may influence both the feasibility and the effectiveness of NFB protocols.

Despite increasing interest in NFB as a non-pharmacological intervention, its application in ID remains insufficiently characterized. Available studies are few and are marked by heterogeneity in participant characteristics, diagnostic definitions, EEG targets, session structure, training duration, outcome measures, and follow-up procedures. Moreover, the methodological quality of the available evidence has not been systematically examined in relation to attention and executive function outcomes in ID.

The aim of this systematic review was therefore to identify and summarize treatment studies evaluating EEG-based neurofeedback in individuals with ID, with a specific focus on attention and executive function outcomes. The review also aimed to compare NFB protocols, evaluate methodological limitations and risk of bias, and derive practical recommendations for the design of future studies.

## 2. Materials and Methods

### 2.1. Review Design

This review was designed as a systematic review of treatment studies evaluating EEG-based neurofeedback in individuals with ID. A systematic search and selection process was used to identify eligible studies, while findings were synthesized narratively because of the limited number of studies and substantial heterogeneity in designs, interventions, populations, and outcome measures. The review was reported in accordance with PRISMA 2020 principles [14]. The completed PRISMA 2020 checklist is provided as Supplementary Material (Supplementary File S1). The review protocol was developed a priori but was not prospectively registered.

### 2.2. Search Strategy

A systematic literature search was conducted in Scopus, PubMed, Embase, the Cochrane Library, and the Web of Science Core Collection. Databases were searched from inception to 12 March 2026. Preliminary searches were conducted in each database using text-based queries designed to identify potentially relevant studies examining the effectiveness of neurofeedback in populations with intellectual and/or neurodevelopmental conditions. The search strings were applied through the standard search interfaces of the respective databases, and no limits were set regarding publication date, language, or document type.

To ensure adequate coverage of the construct of interest, two separate queries were run in Scopus (Elsevier, Amsterdam, Netherlands). The first query (“neurofeedback training and intellectual disability”) retrieved 9 initial records, whereas the second query (“neurofeedback treatment and intellectual disability”) yielded 12. Considering both queries, a total of 21 records were identified; however, only 19 were imported into Rayyan (Rayyan Systems Inc., Cambridge, MA, USA; accessed on 12 March 2026) for the title and abstract screening phases.

Similarly, in Embase (Elsevier, Amsterdam, Netherlands), the following search string was used: (‘neurofeedback/exp OR neurofeedback) AND (‘intellectual impairment’/exp OR ‘intellectual impairment’). This query generated 252 initial records, of which 131 were selected for import into Rayyan. In the Cochrane Library (John Wiley & Sons, Hoboken, NJ, USA), the query (“neurofeedback and ID”) produced 4 records, all of which were imported into Rayyan.

In PubMed (National Center for Biotechnology Information, Bethesda, MD, USA), the search string (“neurofeedback and neurodevelopmental disorders”) returned 453 initial records; however, only 13 were loaded into Rayyan. Finally, in the Web of Science Core Collection (Clarivate, Philadelphia, PA, USA), two queries were performed: the first (“neurofeedback and intellectual disability”) retrieved 60 records, whereas the second (“neurofeedback and neurodevelopmental disorders”) yielded 169. Overall, 229 records were retrieved, of which 100 were imported into Rayyan.

The core search strategy was based on the following structure, shown in Table 1:

The differences observed in the number of records imported across databases reflect the record-handling procedures used prior to screening (e.g., duplicate removal and file preparation for import) and database-specific retrieval and indexing characteristics, including variability in the use and mapping of terms within each database.

Search terms were adapted for each database according to its controlled vocabulary and syntax. Reference lists of eligible full-text articles and relevant reviews were manually screened to identify additional studies.

### 2.3. Eligibility Criteria

Participants: Studies were eligible if they included individuals with ID across childhood, adolescence, or adulthood. ID could be defined according to DSM or ICD criteria, by documented impaired intellectual functioning and adaptive functioning, or by inclusion of genetic or neurodevelopmental conditions typically associated with ID, such as Down syndrome or Fragile X syndrome. Studies including mixed neurodevelopmental samples were eligible only if data from participants with ID could be extracted or clearly interpreted.

Intervention: Eligible interventions were EEG-based NFB protocols. Studies had to provide sufficient information on the NFB procedure, including at least some details on electrode placement, target frequency bands, training goals, session structure, or treatment duration. Non-EEG biofeedback modalities and multimodal interventions were excluded when the specific contribution of EEG neurofeedback could not be isolated.

Comparators: No specific comparator was required. Randomized, controlled, uncontrolled, within-subject, case series, and single-case treatment studies were considered eligible, provided that they reported relevant attention or executive function outcomes.

Outcomes: Eligible studies had to report at least one outcome related to attention or executive functions, including inhibition, working memory, cognitive flexibility, planning, monitoring, impulse control, sustained attention, selective attention, academic task performance, or self-regulation. Outcomes could be assessed using standardized neuropsychological tests, validated clinical or behavioral scales, computerized cognitive tasks, observational behavioral measures, task-performance indices, or EEG-related learning indices.

### 2.4. Exclusion Criteria

Studies were excluded if they involved healthy participants only; included neurodevelopmental disorders other than ID without separable ID data; used interventions other than EEG neurofeedback; lacked attention or executive function outcomes; or consisted only of reviews, editorials, theoretical papers, protocols, or conference abstracts without available full text.

### 2.5. Article Selection

**Figure 1:**
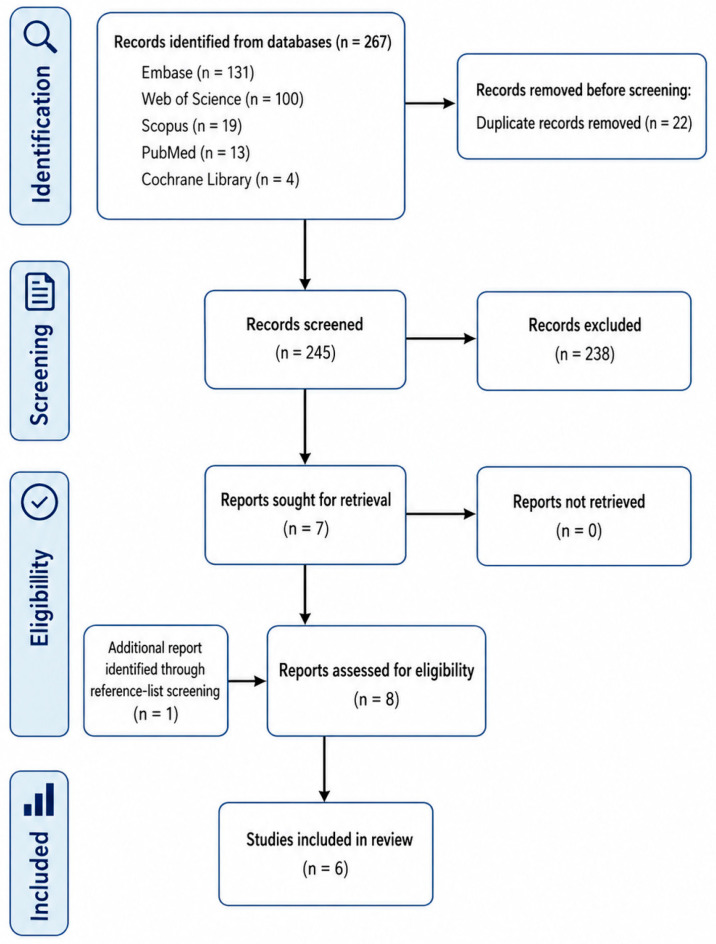
PRISMA 2020 flow diagram of selected studies.

The study selection process, managed using Rayyan [15] and reported according to PRISMA 2020 principles [14], is summarized in Figure 1. The database search identified 267 records: 131 from Embase, 100 from Web of Science, 19 from Scopus, 13 from PubMed, and 4 from the Cochrane Library. After the removal of 22 duplicates, 245 records were screened by title and abstract. Of these, 238 records were excluded because they did not include participants with ID, did not involve EEG neurofeedback, or did not report relevant attention or executive function outcomes. Seven reports were retrieved from the database search, including five potentially eligible full-text treatment reports and two relevant reviews retained for reference list screening. One additional treatment report was identified through reference list screening. Overall, eight reports were assessed in full text, and six treatment studies met the inclusion criteria. Meta-analysis was not performed because of the small number of studies and substantial heterogeneity in study design, intervention protocol, population characteristics, and outcome assessment.

### 2.6. Risk-of-Bias Assessment

Risk of bias was assessed independently by two reviewers. Randomized or controlled trials were evaluated with reference to domains included in the Cochrane risk-of-bias framework (The Cochrane Collaboration, London, UK), including selection bias, performance bias, detection bias, attrition bias, reporting bias, and other sources of bias [16]. Non-randomized and uncontrolled studies were evaluated with reference to ROBINS-I (Bristol, UK) domains where applicable [17]. Because the included studies were heterogeneous in design and several were not randomized trials, results are presented as domain-level qualitative judgments rather than as a summed numerical score.

### 2.7. Data Synthesis

Because the included studies varied markedly in design, sample size, participant characteristics, NFB protocols, outcome measures, and follow-up procedures, quantitative meta-analysis was not appropriate. Findings were therefore synthesized narratively, with emphasis on study characteristics, NFB targets, attention and executive function outcomes, EEG-related outcomes, acceptability, adverse effects, and risk of bias.

Although a larger number of records were identified, many were excluded because they addressed different clinical populations than intellectual disability and/or evaluated cognitive domains/outcomes outside the scope of this review. Consequently, the application of the inclusion criteria substantially reduced the number of studies meeting both population and outcome requirements.

In addition, neurofeedback research specifically targeting intellectual disability is limited, further contributing to the small number of included studies.

## 3. Results

### 3.1. Study Selection

The database search identified 267 records: 131 from Embase, 100 from Web of Science, 19 from Scopus, 13 from PubMed, and 4 from the Cochrane Library. After the removal of 22 duplicates, 245 records were screened by title and abstract. Most records were excluded because they did not include participants with ID, did not involve EEG neurofeedback, or did not report relevant attention or executive function outcomes. Three conference abstracts lacked sufficient available information, and authors were contacted where possible to determine whether full publications were available.

To minimize the risk of publication bias and the omission of unpublished evidence, the review also considered grey literature. In particular, sources such as theses/dissertations were consulted, as well as conference abstracts and conference papers.

Although potentially relevant documents were identified at the initial stage, most of these works were excluded after screening because their content was not consistent with the aim of the review and/or did not fully meet the predefined inclusion criteria.

Five full-text reports were retrieved from the database search. Two relevant reviews were also screened manually, and one additional eligible record was identified through reference list screening. In total, six treatment studies met the inclusion criteria. Meta-analysis was not performed because of the small number of studies and substantial heterogeneity in study design, intervention protocol, population characteristics, and outcome assessment.

### 3.2. Characteristics of Included Studies

The six eligible studies were published between 1982 and 2012 and included children, adolescents, or adults with mild-to-moderate ID or older diagnostic labels corresponding to ID. Some studies included participants with comorbid ADHD-like symptoms, behavioral dysregulation, epilepsy, or other developmental and behavioral difficulties. However, comorbidities were inconsistently reported, and the severity of ID was not always characterized using contemporary diagnostic criteria.

Two studies used randomized or controlled experimental designs [10,13]. Other studies used a tenfold N-of-1 pilot design [8], a clinical case series [12], a single-case report involving twins [9], or a within-subject design [11]. Sample sizes were small across all studies, ranging from two participants in a case report to 23 participants in a clinical case series (Table 2).

### 3.3. Instrumentation Used Across Included Studies

NFB is a form of biofeedback that aims to train self-regulation of brain activity by providing real-time feedback derived from electrophysiological signals.

**Figure 2:**
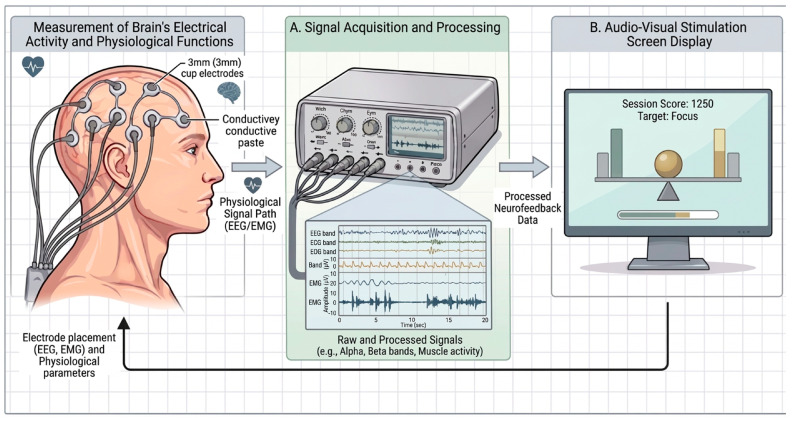
Closed-loop neurofeedback therapy schematic.

Figure 2 illustrates a closed-loop neurofeedback system in which the subject’s neurophysiological activity is measured, processed in real time, and translated into an audiovisual feedback signal aimed at modulating functional parameters (e.g., attentional state/focus). The operational workflow comprises three main phases: acquisition, processing/transformation, and feedback-driven stimulation.

The interaction begins with the placement of electrodes on the scalp (indicated as the electrode configuration). Through this setup, cerebral electrical activity is recorded (typically referred to as EEG/neuroelectrical activity) and, when applicable, associated physiological information is also collected. In this phase, the signal is acquired according to a defined measurement pathway (measurement of brain electrical activity) and subjected to acquisition conditions related to the instrumentation (e.g., electrode-skin contact and signal coupling/conditioning).

The second section of the diagram depicts the signal acquisition and processing unit. The raw signal is:

- Sampled and converted for digital processing;
- Filtered and cleaned to reduce artifacts;
- Transformed into features or indicators (e.g., spectral bands or metrics related to neurophysiological markers) following a multi-channel approach (multi-channel signal processing unit).

The output of this stage is processed neurofeedback data (extracted neurophysiological information), which can be used immediately by the feedback module.

In the third phase, the processed data are used to drive the audiovisual stimulation/display. The diagram highlights an example of feedback conveyed through:

progress/session score indicators,

graphical elements linked to a target (e.g., focus),

optional stimulation/”reinforcement” consistent with the level of the measured neurophysiological parameters.

In this way, the system completes the loop: the outcome of the signal processing (from EEG/features) determines the quality and presentation of the feedback, which in turn influences the subject’s response throughout the ongoing session.

The EEG/neurofeedback systems used in the included studies were as follows:

Fleischman and Othmer [9]: EEG-biofeedback was performed using an EEG Spectrum International system, specifically the Neurocybernetics equipment (Canoga Park, CA, USA).

Hong and Lee [10]: the NFB program system “Neuroharmony M” (Braintech Corp., Seoul, Republic of Korea), designed by the Korea Research Institute of Jungshin Science, was used.

Jackson and Eberly [11]: the apparatus consisted of three main components: (1) an EEG detector and alpha-filtering unit, based on a Grass Model 6 EEG amplifier/recorder to amplify and record brain activity; alpha activity was detected in the 8–13 Hz range, and when alpha was absent, a lamp and counter were activated. The alpha threshold was set to 25% of each participant’s resting alpha (eyes closed). (2) a photocell-based head-orientation monitoring device, consisting of a photodiode placed on a forehead belt oriented toward the screen; the photodiode was connected to an FM transmitter under the participant’s chair and to a receiver in the control room. This device measured head orientation as an index of distraction and did not provide feedback to participants. (3) a visual discrimination apparatus, in which arithmetic problems and visual feedback were projected on a screen through a mirror and a projection tunnel; the display panel included response levers and a green lamp as feedback. The system was controlled by electromechanical relays and a tape programmer.

Breteler et al. [8]: EEG was obtained using a Quickcap and a 40-channel NuAmps amplifier with electrodes placed at FP1, FP2, F7, F3, Fz, F4, F8, FC3, FCz, FC4, T3, C3, Cz, C4, T4, CP3, CPz, CP4, T5, P3, Pz, P4, T6, O1, Oz, and O2.

Thorson and Lipscomb [13]: EEG was collected using a Cyborg Model EEG B541.

Surmeli and Ertem [12]: EEG was acquired with a QEEG/Lexicor system. EEG signals were sampled at 128 samples per second per channel and analyzed using a normative neurometric approach with Nx-Link database software, both pre- and post-treatment.

### 3.4. Neurofeedback Protocols

Hong and Lee [10] used a beta/SMR enhancement protocol designed to increase beta activity in the 16–20 Hz range and SMR activity in the 12–15 Hz range while decreasing theta activity in the 4–7 Hz range. Training was conducted at frontal sites Fp1 and Fp2, with the goal of reducing the theta/beta ratio and improving attention.

Breteler et al. [8] used a structured multi-protocol NFB program in children and adolescents with mild intellectual impairment and ADHD-like behavior. The protocol involved repeated sessions with auditory, puzzle, and video-based feedback components, with EEG recorded from multiple scalp locations.

Surmeli and Ertem [12] used individualized QEEG-guided NFB in a clinical case series of children with mild-to-moderate ID. The protocol involved inhibition of excessive theta and alpha activity at various sites and reinforcement of SMR activity, particularly for hyperactivity and attention-related symptoms.

Fleischman and Othmer [9] reported a case study of twins with mild developmental delay. A 15–18 Hz activation protocol was initially used to increase alertness and cognitive readiness, but emergent anxiety led to a shift toward a 12–15 Hz SMR protocol intended to support calming and physical stability.

Thorson and Lipscomb [13] used a contingent alpha-suppression protocol in adolescents with moderate ID. Participants received feedback when occipital alpha activity was absent, with the aim of increasing oculomotor activity as an index of visual attention.

Jackson and Eberly [11] also used alpha suppression, targeting the 8–13 Hz band during an arithmetic task. Participants received contingent feedback and reinforcement for sustained suppression of alpha activity.

Overall, the protocols differed in target frequencies, electrode sites, feedback modalities, session structure, degree of individualization, and theoretical rationale. This heterogeneity limits direct comparison across studies and precludes firm conclusions regarding the optimal protocol for individuals with ID.

### 3.5. Attention and Executive Function Outcomes

Several studies reported improvements in attention-related outcomes after NFB training, although the measures and designs varied substantially.

Hong and Lee [10] reported that children assigned to the NFB group improved more than those assigned to visual perception training or no-treatment control on the Children’s Color Trails Test Part 2 [18]. The NFB group also showed pre-to-post improvements on the Stroop Color and Word Test [19] and Digit Span of the WISC-R [20], although not all outcomes demonstrated significant between-group differences.

Breteler et al. [8] reported improvements in attention and concentration in a pilot sample of children and adolescents with mild intellectual impairment and ADHD-like symptoms. However, impulse control remained weak, and the non-randomized design, small sample, and absence of a control group limit interpretation.

Surmeli and Ertem [12] reported clinical improvements in most participants on the Developmental Behaviour Checklist [21], WISC-R [20], TOVA [22], and QEEG measures. Improvements were also described in impulsivity, aggression, sociability, academic skills, and other behavioral symptoms. However, the uncontrolled case series design and broad range of reported outcomes make it difficult to attribute improvements specifically to NFB.

Fleischman and Othmer [9] reported increased IQ scores and reduced distractibility in twins after EEG neurofeedback, with apparent maintenance of gains over follow-up. However, because this was a case study without a control condition, results may be influenced by practice effects, maturation, nonspecific intervention effects, or expectancy effects.

Thorson and Lipscomb [13] reported that adolescents receiving contingent feedback significantly reduced alpha density compared with participants receiving non-contingent feedback. The authors interpreted this as evidence that adolescents with moderate ID could learn to modulate EEG activity in a direction relevant to visual attention.

Jackson and Eberly [11] reported reduced alpha activity, improved accuracy on arithmetic tasks, and fewer distractible head-turning responses during the contingency phase compared with baseline. However, the within-subject design and very small sample size limit generalizability.

Across studies, the most consistent finding concerns attention-related outcomes rather than broader executive functions. Evidence for improvements in working memory, inhibition, planning, or cognitive flexibility remains sparse and inconsistent (for more details, see Table 3).

### 3.6. EEG and Neurophysiological Outcomes

EEG-related outcomes generally moved in the direction targeted by each protocol. Hong and Lee [10] reported reductions in theta activity and theta/beta ratio. Breteler et al. [8] reported changes in delta and beta activity toward normative patterns. Surmeli and Ertem [12] described reductions in abnormal QEEG z-scores. Thorson and Lipscomb [13] and Jackson and Eberly [11] reported reductions in alpha activity during alpha-suppression protocols.

However, the relationship between EEG change and clinical or cognitive improvement was not consistently quantified. Few studies reported formal learning curves, time-in-zone measures, amplitude or power change across sessions, or correlations between EEG modulation and behavioral outcomes. Therefore, it remains unclear whether observed cognitive or behavioral changes were mediated by successful EEG self-regulation or by nonspecific features of the intervention.

### 3.7. Acceptability, Adverse Effects, and Follow-Up

Acceptability was incompletely reported. Some studies suggested that NFB was well tolerated, and parents or participants reported positive experiences [8,9]. Thorson and Lipscomb [13] reported generally high acceptability, although four of the initial 20 participants did not complete the study, including two because of a lack of cooperation and two due to scheduling conflicts.

Adverse events were rarely assessed systematically. Surmeli and Ertem [12] reported no adverse effects. Fleischman and Othmer [9] described anxiety emerging after approximately 30 sessions of a 15–18 Hz protocol, which was managed by shifting to an SMR protocol.

This observation is clinically relevant because individuals with ID may be vulnerable to fatigue, frustration, anxiety, sensory overload, or behavioral dysregulation during prolonged or poorly adapted protocols.

Follow-up was variable. Hong and Lee [10] reported maintenance of attention gains at 3 months. Surmeli and Ertem [12] reported maintenance of improvements at 2 years, based mainly on parent reports. Fleischman and Othmer [9] reported long-term maintenance in a case report. Other studies included limited or no follow-up.

### 3.8. Risk of Bias

Overall, the risk of bias was moderate to high across the available literature. Common limitations included small sample size, absence of adequate allocation concealment, lack of sham or active control procedures, absence of participant or assessor blinding, reliance on subjective or caregiver-reported outcomes, incomplete reporting of comorbidities, and limited reporting of attrition, adverse events, and effect sizes.

The randomized or controlled studies provide the strongest available evidence, but they remain limited by small samples and incomplete blinding [10,13]. Uncontrolled studies, case series, and single-case reports provide useful feasibility and hypothesis-generating information but cannot establish efficacy. The overall certainty of evidence should therefore be considered low to very low.

## 4. Discussion

### 4.1. Principal Findings

This systematic review identified six treatment studies evaluating EEG neurofeedback for attention or executive function-related outcomes in individuals with ID. Overall, the evidence suggests that EEG neurofeedback may be feasible and may improve attention-related measures in some individuals with ID. However, the available evidence remains preliminary. The low number of included studies can be explained by two main factors. First, many of the records retrieved during the search were excluded because they involved different clinical populations than intellectual disability and/or assessed cognitive abilities/outcomes outside the scope of this review. Second, the neurofeedback literature specifically targeting individuals with ID is currently limited. The literature is limited by small samples, heterogeneous populations, variable diagnostic characterization, diverse NFB protocols, inconsistent outcome measures, scarce use of sham or active-control conditions, limited blinding, and incomplete reporting of effect sizes and adverse events.

The most consistent findings concern attention-related outcomes, including sustained attention, task accuracy, distractibility, or attention-related neuropsychological measures. Evidence for broader executive functions, such as working memory, inhibition, planning, and cognitive flexibility, is less robust and should be interpreted with caution.

### 4.2. Clinical Relevance for Intellectual Disability

Attention and executive function difficulties are clinically important in individuals with ID because they may interfere with learning, adaptive behavior, communication, social functioning, and autonomy [23]. Interventions that improve attentional regulation or behavioral self-control could therefore have meaningful downstream effects. NFB is conceptually attractive because it targets self-regulation of neural activity and can be adapted through game-like feedback, individualized thresholds, and gradual shaping of performance.

Nevertheless, ID presents specific challenges for NFB implementation. Participants may have reduced ability to understand task instructions, limited sustained engagement, sensory sensitivities, variable motivation, and comorbid conditions that affect EEG signals or behavioral outcomes. These characteristics highlight the need for protocols that are developmentally appropriate, tolerable, individualized, and carefully monitored.

### 4.3. Protocol Heterogeneity and Mechanistic Uncertainty

The reviewed studies used markedly different NFB approaches, including beta/SMR enhancement, theta reduction, QEEG-guided individualized training, alpha suppression, and sequential protocol adjustment. This heterogeneity reflects the exploratory nature of the field but also limits conclusions about which NFB targets are most appropriate for ID.

SMR and beta enhancement protocols are often intended to promote alertness, attention, and sensorimotor regulation, whereas theta reduction or theta/beta ratio protocols are frequently used to target inattention and underarousal. Alpha suppression protocols have historically been used to increase task engagement or reduce internally focused idling states during cognitive performance. QEEG-guided protocols attempt to individualize training according to baseline EEG deviations. However, the reviewed studies rarely tested whether successful EEG self-regulation mediated behavioral improvement. Therefore, the mechanisms through which NFB might improve attention in ID remain uncertain.

Future studies should report not only pre-to-post behavioral outcomes but also learning indices during training, such as amplitude or power change, time-in-zone, session-by-session learning curves, and correlations between EEG modulation and clinical response. Without such data, it remains difficult to distinguish specific neurofeedback effects from nonspecific effects related to attention from therapists, repeated practice, reinforcement, motivation, expectancy, or structured cognitive engagement.

### 4.4. Risk of Bias and Certainty of Evidence

The overall certainty of evidence is low to very low. Even the relatively stronger controlled studies are limited by small sample size, incomplete blinding, and limited reporting of allocation concealment or sham procedures. Uncontrolled case series and case reports provide useful feasibility information but cannot establish efficacy.

A particular limitation is the scarcity of active or sham-controlled designs. This is important because NFB interventions include several nonspecific therapeutic components, such as repeated therapist contact, structured sessions, gamified reinforcement, motivational feedback, and expectancy effects. Without adequate comparators, improvements cannot be confidently attributed to EEG-contingent learning.

Another limitation is incomplete reporting of effect sizes. Statistical significance alone is insufficient, especially in small samples. Future trials should report standardized effect sizes, confidence intervals, responder analyses, and clinically meaningful change thresholds.

### 4.5. Acceptability and Safety

Acceptability and safety deserve greater attention in this field. NFB is often presented as non-invasive and low risk, but this should not lead to underreporting of adverse events. Individuals with ID may experience fatigue, frustration, anxiety, sensory discomfort, behavioral dysregulation, or reduced tolerance for prolonged sessions. The anxiety observed in one case report after a higher-frequency activation protocol illustrates the importance of monitoring arousal and emotional state during treatment.

Future studies should include systematic reporting of dropout, session completion, cooperation, adverse events, caregiver satisfaction, participant enjoyment, and reasons for discontinuation. These outcomes are especially relevant when evaluating feasibility in clinical ID populations.

### 4.6. Implications for Future Trials

The next generation of studies should move beyond proof-of-concept reports and adopt rigorous clinical-trial methodology. Adequately powered randomized controlled trials are needed, ideally comparing EEG-contingent NFB with sham feedback, active cognitive training, alternative frequency-band training, or treatment as usual. Blinded assessment should be used whenever possible. Participant characterization should include ID severity, adaptive functioning, comorbid ADHD or autism symptoms, epilepsy, sleep problems, medication status, and baseline EEG features.

Outcomes should be selected a priori and should include validated measures of attention and executive functions. Because caregiver and teacher reports may be influenced by expectancy, objective neuropsychological or computerized measures should be combined with ecologically valid behavioral assessments. Longer follow-up is needed to determine whether gains are maintained and whether they generalize to daily functioning (Table 4).

### 4.7. Limitations of This Review

This review has limitations. First, the evidence base is very small, with only six eligible treatment studies. Second, older studies used historical terminology and diagnostic practices that do not fully align with contemporary definitions of ID. Importantly, this concern applied to only two of the included studies, for which we explicitly mapped historical diagnostic labels (e.g., “mental retardation”) to ID consistent with DSM-5-TR as operationalized in our eligibility criteria (i.e., impaired intellectual and adaptive functioning and/or the inclusion of genetic/neurodevelopmental conditions typically associated with ID). Third, study heterogeneity prevented meta-analysis. Fourth, some studies provided incomplete information on comorbidities, intervention parameters, adverse events, and effect sizes. Finally, although multiple databases and reference lists were searched, unpublished studies or poorly indexed reports may have been missed.

Despite these limitations, this review provides a structured synthesis of a small but clinically relevant literature and identifies key methodological requirements for future research.

## 5. Conclusions

EEG neurofeedback may be feasible and potentially beneficial for attention-related difficulties in some individuals with intellectual disability. However, current evidence remains preliminary and does not yet support routine clinical implementation outside structured clinical or research settings. The available studies are limited by small sample sizes, heterogeneous protocols, variable outcome measures, limited blinding, insufficient sham-controlled evidence, and incomplete reporting of effect sizes and adverse events.

Future research should prioritize adequately powered randomized controlled trials with transparent protocol reporting, active or sham comparators, blinded outcome assessment, standardized attention and executive function measures, systematic learning indices, careful adverse event monitoring, and longer follow-up. Such studies are needed to determine whether EEG neurofeedback has specific, durable, and clinically meaningful effects in individuals with intellectual disability.

## Figures and Tables

**Table 1:** Records initially retrieved by the database and search string.

Database	Query	Applied Research	Record Initially Searched
Scopus	“Neurofeedback training and Intellectual Disability”	Standard text search in the database	9
Scopus	“Neurofeedback treatment and intellectual disability”	Standard text search in the database	12
Embase	(‘neurofeedback/exp OR neurofeedback) AND (‘intellectual impairment’/exp OR ‘intellectual impairment’)	Standard text search in the database	252
Cochrane Library	“Neurofeedback and ID”	Standard text search in the database	4
Pubmed	“Neurofeedback and neurodevelopmental disorders”	Standard text search in the database	453
Web of Science Core Collection	“Neurofeedback and intellectual disability”	Standard text search in the database	60
Web of Science Core Collection	“Neurofeedback and neurodevelopmental disorders”	Standard text search in the database	169

No date limits, no language restrictions, and no publication-type filters were applied in any database. “Record initially searched” refers to the number of records retrieved using the specified search strings prior to deduplication and screening.

**Table 2:** Main characteristics of included treatment studies.

Study	Design	Participants and Diagnosis	Neurofeedback Protocol	Main Outcomes
Hong and Lee [10]	Randomized controlled experimental study	21 elementary school children with mild ID, assigned to NFB, visual perception training, or control groups. Mild ID; comorbidities not clearly reported.	Beta/SMR up-training and theta reduction at Fp1/Fp2; reduction in theta/beta ratio.	CCTT-2, Stroop Color and Word Test, Digit Span, theta amplitude, beta/SMR amplitude, theta/beta ratio.
Breteler et al., 2012 [8]	Tenfold N-of-1 pilot design	Children and adolescents aged 9–18 years with IQ 50–85 and ADHD-like behavior. Mild intellectual impairment/borderline intellectual functioning with ADHD-like symptoms.	Multi-component NFB program with auditory, puzzle, and video feedback; multichannel EEG recording.	Amsterdam Neuropsychological Tasks, Bourdon-Vos, EEG measures, satisfaction.
Surmeli and Ertem, 2010 [12]	Clinical case series	23 children aged 7–16 years. Mild-to-moderate ID; several behavioral and neurological comorbidities reported.	QEEG-guided theta/alpha inhibition and SMR reinforcement at individualized sites.	WISC-R, TOVA, Developmental Behaviour Checklist, QEEG, parent-reported behavioral outcomes.
Fleischman and Othmer, 2006 [9]	Case study	Twin sisters aged 8 years and 5 months. Mild developmental delay with attention, behavioral, anxiety, sleep, and social difficulties.	Initial 15–18 Hz activation protocol followed by 12–15 Hz SMR protocol after anxiety emerged.	WISC-III, ADHD symptom checklists, parent reports, long-term follow-up.
Thorson and Lipscomb, 1982 [13]	Randomized controlled experimental study	16 adolescents with moderate ID completed the study. Moderate ID and attention deficits; participants on CNS depressants or anticonvulsants excluded.	Occipital alpha suppression; contingent feedback for absence of 9.5–10.5 Hz alpha activity.	Occipital alpha density as an index of EEG modulation/visual attention.
Jackson & Eberly, 1982 [11]	Within-subject design	5 adults with ID. ID according to historical terminology; low attention and high distractibility.	Alpha suppression in the 8–13 Hz band during arithmetic task.	Alpha activity, arithmetic accuracy, number of problems completed, distractible head-turns.

Abbreviations: ID, intellectual disability; NFB, neurofeedback; SMR, sensorimotor rhythm; ADHD, attention-deficit/hyperactivity disorder; CCTT-2, Children’s Color Trails Test-Part 2; WISC-III, Wechsler Intelligence Scale for Children, 3rd edition; WISC-R, Wechsler Intelligence Scale for Children-Revised; TOVA, Test of Variables of Attention; QEEG, quantitative electroencephalogram; CNS, central nervous system.

**Table 3:** Summary of findings and methodological limitations.

Study	Main Findings	Follow-Up	Acceptability/ Adverse Events	Main Limitations
Hong and Lee [10]	The NFB group improved on CCTT-2 compared with visual perception training and control groups; within-group improvements in Stroop and Digit Span; reductions in theta activity and theta/beta ratio.	3-month maintenance reported.	No specific adverse event data reported.	Small sample; limited blinding; no sham control; incomplete effect-size reporting.
Breteler et al. [8]	EEG changes toward normative patterns; attention and concentration reportedly improved, but impulse control remained weak.	Not clearly systematic.	Children reported improved perception of their situation.	Non-randomized design; no control group; small sample; limited blinding.
Surmeli and Ertem [12]	Most participants reportedly improved on behavioral, cognitive, attention, and QEEG measures.	2-year parent-reported stability.	No adverse effects reported.	Uncontrolled case series; convenience sampling; broad outcomes; expectancy and maturation effects possible.
Fleischman and Othmer [9]	IQ increases and reduced distractibility reported in twins.	Long-term maintenance reported.	Anxiety emerged after activation protocol and improved after protocol adjustment.	Case study; no control group; practice effects possible; no blinded assessment.
Thorson and Lipscomb [13]	The contingent-feedback group reduced alpha density more than the non-contingent-feedback group.	Not clearly reported.	Four participants did not complete the study; generally acceptable among completers.	Small sample; allocation concealment unclear; incomplete blinding; 20% attrition.
Jackson and Eberly [11]	Alpha activity decreased; arithmetic accuracy improved; distractible head-turns decreased.	Not reported.	Not reported.	Very small sample; within-subject design; no control group; possible practice effects.

Abbreviations: CCTT-2, Children’s Color Trails Test-Part 2; NFB, neurofeedback; QEEG, quantitative electroencephalogram.

**Table 4:** Recommended methodological standards for future NFB studies in intellectual disability.

Domain	Recommendation
Study design	Use adequately powered randomized controlled designs whenever feasible.
Comparator	Include sham NFB, active control, or alternative frequency-band control conditions.
Blinding	Use blinded outcome assessors and, when possible, blinded participants/caregivers.
Participants	Define ID using contemporary diagnostic criteria, IQ/adaptive-function measures, and severity level.
Comorbidities	Systematically report ADHD, autism, epilepsy, sleep disorders, medications, and behavioral comorbidities.
Protocol reporting	Report electrode sites, target frequencies, reward/inhibit bands, thresholds, session duration, number of sessions, and feedback modality.
Learning indices	Report within-session and across-session learning curves, power/amplitude changes, time-in-zone, and responder status.
Outcomes	Predefine primary attention or executive function outcomes and use validated neuropsychological or behavioral measures.
Safety and feasibility	Record adverse events, anxiety, fatigue, frustration, sensory intolerance, dropout, and caregiver/participant acceptability.
Follow-up	Include follow-up assessments of at least 3–6 months when feasible.
Reporting	Provide effect sizes, confidence intervals, missing-data handling, and protocol deviations.

Abbreviations: ADHD, attention-deficit/hyperactivity disorder; ID, intellectual disability; NFB, neurofeedback.

## Data Availability

The data presented in this study are available on request from the corresponding author due to the nature of the extracted study-level datasets generated during the screening and data extraction process, which are not publicly hosted but can be shared after verification of the requester’s purpose.

## Supplementary Materials

The following supporting information can be downloaded at: https://www.mdpi.com/article/10.3390/jcm15134947/s1, File S1: PRISMA 2020 checklist.

## Abbreviations

The following abbreviations are used in this manuscript:

ID	Intellectual Disability
EFs	Executive Functions
NFB	Neurofeedback
SMR	Sensorimotor rhythm
ADHD	Attention-Deficit/Hyperactivity Disorder
DSM	Diagnostic and Statistical Manual of Mental Disorders
ICD	International Classification of Diseases
CCTT-2	Children’s Color Trails Test—Part 2
SCWT	Stroop Color and Word Test
WISC-III	Wechsler Intelligence Scale for Children (3rd ed.)
MMR	Mild Mental Retardation
BR	Borderline Retardation
ANT	Amsterdam Neuropsychological Testing Program
DSM-IV	Diagnostic and Statistical Manual of Mental Disorders—Fourth Edition
WISC-R	Wechsler Intelligence Scale for Children—Revised
TOVA	Test of Variables of Attention
DBC-P	Developmental Behaviour Checklist—Parent/Carer version
QEEG	Quantitative Electroencephalogram
DSM-III-R	Diagnostic and Statistical Manual of Mental Disorders—Third Edition—Revised
CNS	Central Nervous System
PPVT	Peabody Picture Vocabulary Test
ANCOVA	Analysis of Covariance
